# Surgical health policy advocacy and suicide prevention: the San Diego-Coronado bridge case

**DOI:** 10.1186/s40621-026-00671-8

**Published:** 2026-03-30

**Authors:** John R. Austin, Joy H. Song, Laura N. Haines, Allison E. Berndtson, Jessica L. Weaver, Jarrett E. Santorelli, Jeanne G. Lee, Leslie M. Kobayashi, Laura M. Adams, Alan Smith, Jay J. Doucet, Amy E. Liepert

**Affiliations:** 1 Division of Trauma, Surgical Critical Care, Burns and Acute Care Surgery, Department of Surgery, University of California San Diego, 200 W. Arbor Drive, MC 8896, San Diego, CA 92103 USA; 2 Department of Surgery, Temple University, Philadelphia, PA 19140 USA

**Keywords:** Surgical advocacy, Suicide, Injury prevention, Bridge suicide, Suicide prevention

## Abstract

**Introduction:**

The 1969 San Diego–Coronado Bridge (SDC bridge) has been the site of more than 473 suicides by jumping. In 2019, a “bird spike” barrier was installed along road edge walls as a suicide deterrent. We hypothesize that current anti-suicide measures have been ineffective in reducing the annual suicide-by-jumping rate at the SDC bridge.

**Methods:**

The medical examiner and UCSD trauma registries were reviewed for suicide and attempted suicide following jumping from the SDC bridge from January 1997 to August 2024. The data collected included demographics, the injury severity score (ISS), toxicology, and the home area deprivation index (ADI). The suicide rates for the SDC bridge compared with those for the entire county from 1997 to 2018 and 2019 to 2024, before and after the installation of the “bird spike” barrier, were analyzed via Poisson rate tests and difference-in-differences regression. Potential years of life lost (PYLL) and incremental cost-effectiveness ratios (ICERs) were also calculated.

**Results:**

There were 10,411 suicide deaths in San Diego County and 298 people who jumped from the SDC bridge from January 1997 through August 2024. Among these bridge jumps, 272 (91.2%) resulted in death after jumping from the SDC bridge (8 at the hospital; 264 at the scene), whereas 26 survivors (8.7%) were recorded. The ISS of survivors was significantly lower than that of those who died (18 ± 10 vs. 42 ± 7, *p* < 0.001). The rate of bridge suicide attempts remained unchanged before and after the deterrent bird spike was installed (pre: 227 over 22 years, post: 71 over 6 years, *p* = 0.32). DiD analysis revealed no difference between SDC bridge suicides and county jump-from-height suicides (DiD Coeff. <0.0001, *p* = 0.973).

**Conclusion:**

A costly project to install a bird spike suicide barrier on the SDC bridge did not decrease the number of bridge suicides. A fence-style physical barrier supported by surgeon-led advocacy could be highly cost-effective. Ongoing advocacy efforts by trauma surgeons and their allies with policymakers are making progress toward implementing effective preventative measures to reduce SDC bridge suicides.

**Level of Evidence III:**

Prognostic and Epidemiological, Case Control Study without Negative Criteria.

## Introduction

A “suicide bridge” is a bridge often used by people to end their lives, usually by jumping into the water or onto the ground below. Compared with other methods, suicide prevention advocates believe that suicides by bridges are more likely to be impulsive or unplanned and that installing effective barriers can significantly reduce the number of bridge suicides [1]. 

The San Diego–Coronado (SDC) bridge is a 2.1-mile-long (3.4 km), 200-foot-high (61 m) structure connecting the cities of San Diego and Coronado, California. Its impressive span and height have unfortunately made it an attractive landmark and a site for suicide attempts throughout its history [2]. Since opening in 1969, approximately 473 SDC bridge suicides have occurred, making it the second most commonly used U.S. suicide bridge after the Golden Gate Bridge [3]. Since the construction of suicide barriers on the San Francisco Bay Golden Gate Bridge (SFBGG) in 2023, the SDC bridge has become the U.S. bridge with the highest annual rate of suicide-by-jumping, but it still lacks an effective suicide barrier.

**Fig. 1:**
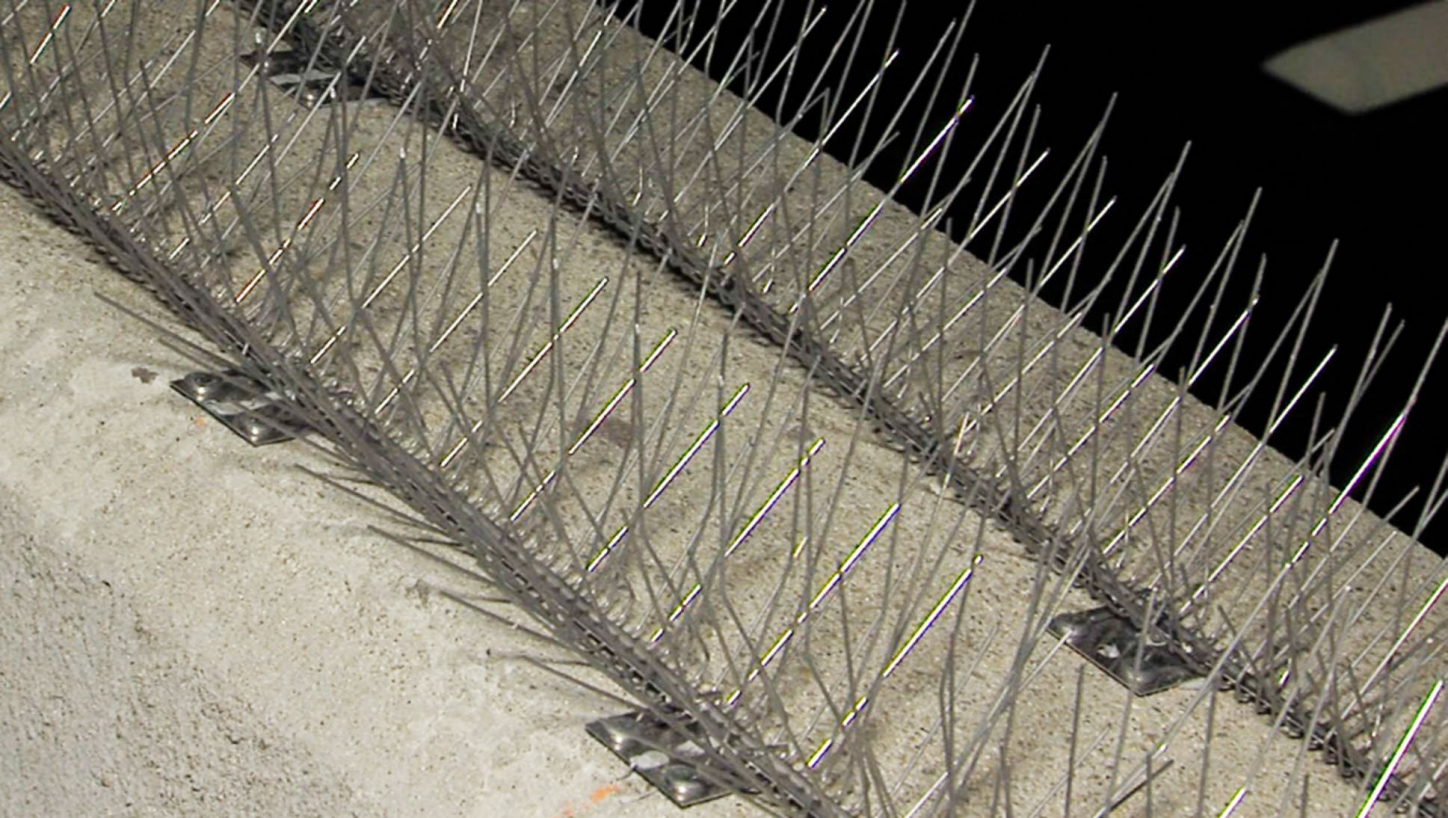
Nixalite™ Bird Exclusion “bird spike” system was installed on the San Diego-Coronado Bridge in 2019 as a visual, psychological suicide prevention deterrent

There are many strategies to prevent suicide, including routine depression screening, youth education and awareness, cognitive‒behavioral therapy, electronic health record screening, and means restrictions. Among these, limiting access to lethal means consistently provides strong evidence of reducing the number of suicides [4–6]. In 2008, the National Suicide Prevention Lifeline released white paper stating that bridge barriers are the most effective way to prevent suicide [7]. A 2020 Cochrane Database Systematic Review and Meta-analysis of 12 studies revealed that at sites where restriction techniques such as barriers were used, either alone or with other methods, the number of suicides decreased by 91% [8]. Although suicide prevention signage with a 1–800 suicide phone number is posted on the SDC bridge, no effective physical barrier has yet been installed. Suicide prevention call boxes were placed on the SDC bridge but were removed in 2008 because they were seen as ineffective. Members of the public, health advocates, and policymakers at both the state and local levels have repeatedly urged Caltrans, California’s highway management division, to install a barrier since the first SDC bridge suicide-by-jumping occurred in 1969. Caltrans proposed a permanent suicide barrier in 2018, estimated to cost $140 million, but this did not find funding [3]. In 2019, a temporary “bird spike” barrier with 4-inch metal spikes was installed on top of the roadside bridge’s 4-foot wall as a suicide deterrent at an expense of $420,000 [3]. This barrier remains in place six years later (Fig. 1).

In the context of surgical health policy advocacy, we hypothesized that previous antisuicide measures have been ineffective in reducing the annual bridge suicide rate compared with San Diego County suicide rates. We also hypothesized that the costs associated with potential years of life lost (PYLL) and the value of life lost greatly exceed the costs of implementing effective restrictions on suicide on the SDC bridge. A secondary goal is to identify strategies to support fundable solutions for deterring suicide-by-jumping on the SDC bridge.

## Methods

We collected retrospective data from January 1997 to August 2024, assessing suicides from the San Diego Medical Examiner and suicide attempts from the San Diego-Coronado (SDC) Bridge brought to our urban Level 1 Trauma Center through our trauma registry. The entire SDC bridge is physically within our center’s catchment. At our level 1 trauma center, all suicide survivors receive post-intervention strategies, including inpatient psychiatric and social worker assessment and follow-up. All survivors received blood alcohol and urine drug screening, and those who are positive also receive a social worker screening, brief intervention, and possible referral for substance abuse (SBIRT).

Annual deaths were recorded for all suicides in the county and for suicides and survivors jumping from the SDC bridge. The “jumper” group was defined as all individuals injured or killed after jumping from the SDC bridge and documented in the County Medical Examiner database and/or the Level 1 Trauma Center registry. Data on age, sex, race, injury severity score (ISS), and home ZIP codes, along with the National Area Deprivation Index (ADI) percentile, were collected. The area deprivation index (ADI) is a validated, census-based measure of neighborhood socioeconomic disadvantage that incorporates factors such as income, education, employment, and housing quality. Developed to quantify social determinants of health at the neighborhood level, the ADI enables comparisons of relative deprivation across geographic areas (e.g., census block groups). Higher ADI scores indicate greater socioeconomic disadvantage and are associated with poorer health outcomes and reduced access to healthcare resources [4]. Median driving distances from residence to jumping event Zip Code centroids were determined and mapped using Origin-Destination Cost Matrix network analysis in ArcGIS Pro.

Comparison of SDC bridge rates versus all suicide rates for two five-year periods (2013–2018 vs. 2019–2024), before and after the completion of the “bird spike” barrier, was performed via Poisson rate tests and difference-in-differences (DiD) analysis. Potential years of life lost (PYLL) were calculated by subtracting the age at death from an arbitrarily chosen standard age of 65 years and then summing individual YPLL. The value of life lost was estimated via the central value of the value of a statistical life year lost (VSLY) from the Department of Health and Human Services (HHS) to evaluate the potential cost-effectiveness of suicide, which means restriction interventions for the population of suicide victims at the SDC bridge [5, 6]. 

We calculated annual estimates of the PYLL and costs of SDC bridge suicides since the bridge’s construction in 1969. This included a project cost of $140 million for a perfect suicide barrier, a 33-year project lifespan, and valuing each life year at $495,000. We used these figures to determine the incremental cost-effectiveness ratio (ICER) per VSLY for a barrier with a reported effectiveness of the SFGBB compared to the SDC bird spike barrier. For the SDC bridge suicides, a conservative standard age of 65 years was used, and the average and total potential years of life lost (PYLL) per death were calculated. With an estimated value of a statistical life year (VSLY) of $495,000 (from HHS 2024), we determined the total VSLY lost due to jumping at the San Diego-Coronado Bridge since 1997.

Categorical values were analyzed by chi-square analysis, and means were compared via ANOVA. IBM SPSS Statistics version 30, Armonk, NY, was used for analysis. Geospatial analysis was performed with ArcGIS Pro 3.6.0, (esri, Redlands, CA). This study was conducted under UC San Diego Institutional Review Board Study Approval #151,611.

## Results

There were 10,411 suicide deaths in San Diego County and 298 individuals who jumped from the San Diego–Coronado Bridge between January 1997 and August 2024. Among these bridge jumpers, 272 (91.2%) died after jumping (8 at the hospital; 264 at the scene), whereas 26 survived to hospital discharge (8.7%). Survivors were younger (29 ± 11 years vs. 37 ± 14 years, *p* < 0.001), with no significant difference in sex (69.2% male vs. 72.8% male, *p* = 0.61) or in the percentage of white patients (73.1% vs. 68.4%, *p* = 0.79). The injury severity score (ISS) among survivors was lower than that among those who died after admission (18 ± 10 vs. 42 ± 7, *p* < 0.001). The bridge jumper area deprivation index (ADI) was significantly lower than the county average ADI (17% ± 10% vs. 9%, *p* < 0.001) (Table 1).

The rate of bridge suicide attempts remained consistent from 1997 to 2018 compared with 2019–2024, both before and after the installation of the deterrent system, as shown by the Poisson rate test (pre: 227 attempts over 22 years; post: 71 attempts over 6 years; *p* = 0.32). The trend of completed bridge suicides also did not differ from the county’s overall suicide trend (*p* = 0.56).

**Fig. 2:**
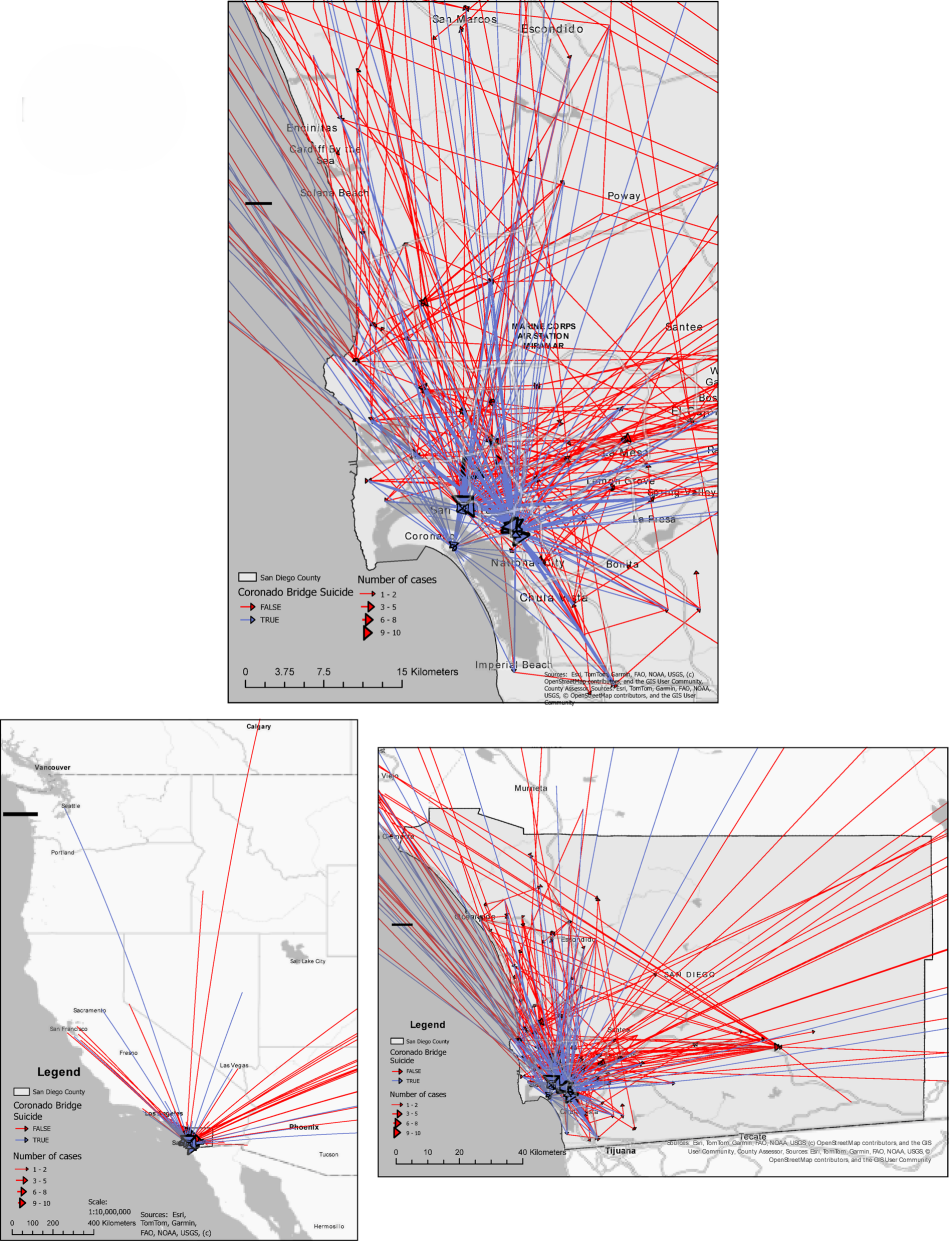
Straight line distance from residence to event Zip Code centroids at downtown, county, and state ranges

There was no significant difference in residence county ZIP codes between jumping sites, 36 of 281 (12.8%) attempts at the SDC bridge with known ZIP codes were from outside San Diego County, compared to 105 of 551 (19.1%, *p* = 0.08) jumping from non-SDC bridge locations. Median driving distance from residence ZIP centroid to event ZIP centroid location was higher in SDC bridge jumping than from other locations (18.3 km, IQR 10–30 vs. 10.7 km, IQR 0–32, *p* < 0.0001 by Mann-Whitney U test) (Table 2). Straight line distance from residence to event Zip Code centroids is shown in Fig. 2 at downtown, county, and state ranges.

**Fig. 3:**
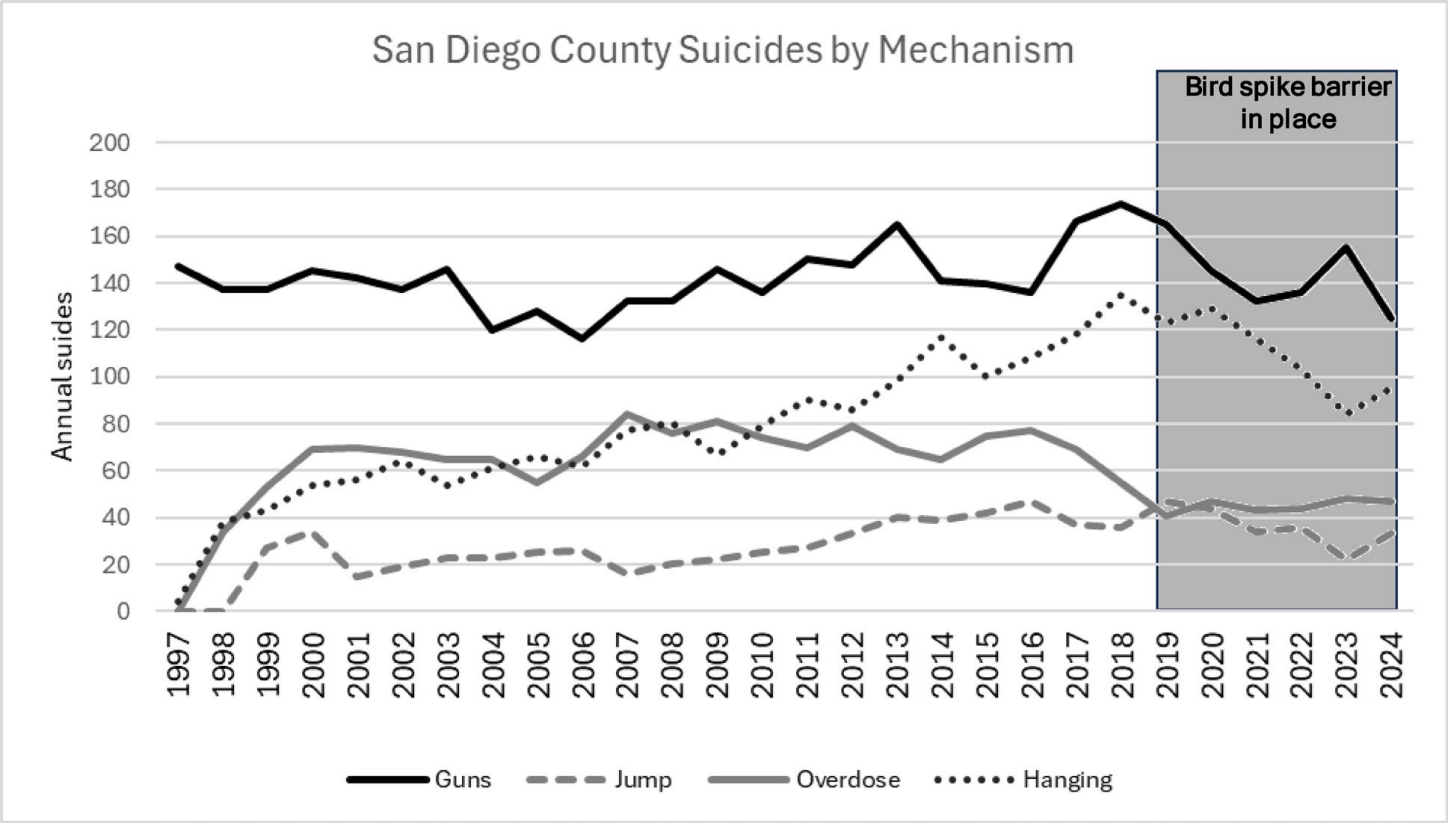
Annual San Diego–Coronado Bridge Suicide and All County suicide events from 1997–2024

**Fig. 4:**
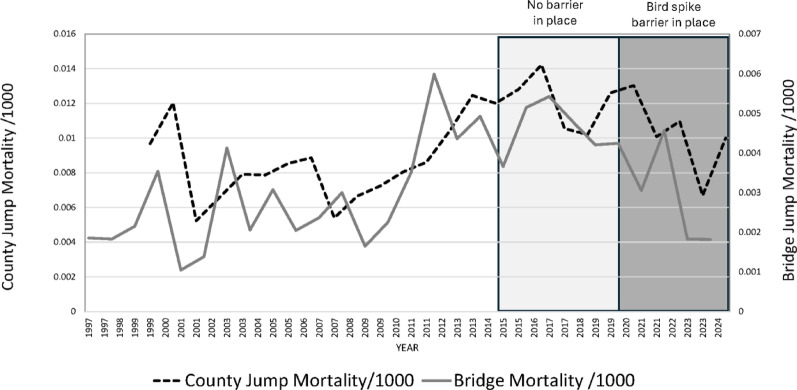
Annual San Diego–Coronado Bridge Suicide versus other County Suicides from height rates/1000 population, showing the Bird Spike Barrier Period and difference-in-differences periods

Significant fluctuations in common methods of suicide were observed in San Diego County during the study period, including peak periods of the SARS-CoV-2 pandemic (Fig. 3). However, difference-in-differences (DiD) analysis of the county suicide rate for jumps from height to completed bridge suicides revealed no significant difference between the pre- and postintervention periods (DiD coefficient: <0.0001, 95% CI: -0.01–0.04, *p* = 0.973; Fig. 4).

The (PYLL) per death was 28 years, totaling 8,344 PYLLs or approximately 309 PYLLs annually. The total VSLY lost due to jumping at the San Diego-Coronado Bridge since 1997 is approximately $4.1 billion. Extending this estimate to include all 473 SDC bridge suicides since the bridge’s construction in 1969, the total loss is approximately $8.1 billion. Assuming a project cost of $140 million for a perfect suicide barrier, which would theoretically prevent all suicides, over a 33-year project lifespan, and valuing each life year at $495,000, the incremental cost-effectiveness ratio (ICER) would be $13,730 per VSLY compared with the bird spike barrier [7–9]. 

**Table 1:** Characteristics and outcomes of San Diego–Coronado Bridge jumpers (1997–2024). Values are mean ± SD or n (%)

Table one	Survivors (*n* = 26)	Died after jump (*n* = 272)	*p*-value
Age, years	29 ± 11	37 ± 14	< 0.001*
Male sex	18 (69.2%)	198 (72.8%)	0.61
White race	19 (73.1%)	186 (68.4%)	0.79
Mean Injury Severity Score (ISS)^+^	18 ± 10	42 ± 7	< 0.001*

*P < 0.05

^+^ISS comparison limited to patients transported to hospital

**Table 2:** Comparison of San Diego–Coronado Bridge suicide attempts with other jumping locations in San Diego County

Table two	Coronado Bridge	Other jumping locations	*p*-value
Residence outside San Diego County	36/281 (12.8%)	105/551 (19.1%)	0.08
Median Driving Distance residence ZIP centroid → event ZIP centroid, km	18.3 (IQR 10–30)	10.7 (IQR 0–32)	< 0.0001*

*P < 0.05

## Discussion

The rate of suicide-by-jumping at the SDC bridge did not change after installing a bird spike deterrent strip. This ineffective, underinformed public policy decision—made without significant input from healthcare stakeholders or experts—cost $420,000 and has failed to reduce the suicide rate since its installation. Witnesses reported that the spike barrier was easily covered with a car floormat or simply ignored by those attempting to jump. Within two weeks of completing the $420,000 bird spike project in 2019, three people still managed to commit suicide from the SDC bridge over a 24-hour span. The subsequent suicide rate for the next two years did not significantly differ from the county’s overall trend, as confirmed by DiD analysis. Our estimates of PYLL and the value of life lost by victims of these suicides are substantial, mainly due to the victims’ young age, which represents a significant loss to society in both human and economic terms.

The SFBGG bridge has historically been the most common site for bridge suicides worldwide, with over 1,700 deaths since 1937. After decades of resistance, such as that encountered by the SDC bridge, a steel grid net was installed 20 feet below the SFGGB deck in 2024, reducing suicides by 73% [7]. Given a nominal 73% effectiveness rate, the adjusted ICER would be $18,315. The World Health Organization (WHO) states that an intervention is cost-effective if it costs up to three times the per capita GDP to save one life year. In 2023 dollars, this is $248,308 for the USA. Compared to an adjusted ICER of $18,315, we have demonstrated that a barrier as effective as the SFGGB barrier would be very cost-effective.

Historically, bridges were not built with suicide deterrence in mind, and current federal standards do not include suicide prevention criteria. However, evidence supporting the effectiveness of physical deterrents such as nets and barriers on bridges, such as the SFBGG, is increasing awareness of this type of suicide prevention. Since 2021, bills such as H.R.3505—the ‘Barriers to Suicide Act’—have been introduced to promote the installation of evidence-based deterrents on bridges [10]. Suicide deterrence as a funded design feature in all future high-level bridges and structures is a worthwhile advocacy goal.

Restricting access to means of suicide is effective. Many self-harm attempts are impulsive acts made during intense but brief personal crises. Individuals experiencing suicidal thoughts often turn to the most easily available methods. By limiting access, especially to highly lethal means such as jumps from heights, attempts can be discouraged, and the immediate crisis can subside without leading to irreversible consequences [12]. Meanwhile, individuals who carefully choose their method beforehand are often influenced by how accessible the method is and how well known it is within their sociocultural context [13]. Consequently, if access to a specific method is restricted, these individuals may be forced to reconsider or select a more readily available but less lethal method of suicide alternative [12, 14]. Additionally, this “substitute effect” of at-risk individuals choosing an alternative method of suicide may be overstated, with the effect generally being small and observed over longer periods at the whole-population level [15, 16]. 

Prominent locations, such as bridges, can develop a reputation as places linked to suicide attempts because of publicized incidents and media coverage [11, 12]. This visibility can attract more individuals to these locations for similar actions, and measures such as restricting access can reduce this risk. There was not a significant difference in home ZIP codes by county, but a significant number of persons did travel longer distances before jumping from SDC bridge, up to 4237 km. Unfortunately, we cannot determine any differences in the overall intent, whether bridge suicide attempts were impulsive or more planned by those visiting San Diego County. Effective physical barriers are essential for bridges or high places because they provide effective protection. Trauma surgeons and healthcare providers see recurring injury patterns in their communities, which can foster hopelessness in providers if there is no potential for change. The American College of Surgeons Committee on Trauma requires Level 1 trauma centers to have an injury prevention program that is cost-effective, feasible, fundable, and publicly acceptable [13]. 

Trauma surgeons can use clinical data to enhance care and prevent injuries while also focusing on clinician wellness and public health policy advocacy. Since 1985, concerns about fatalities on the SDC bridge have increased, with efforts to install a suicide barrier beginning in approximately 2015. Advocacy by mental health professionals and trauma surgeons led to the creation of California Senate Bill SB656, “San Diego-Coronado Bridge: physical suicide deterrent system,” in 2019. This bill established a stakeholder committee, including a trauma surgeon, to discuss the need for and the impact of a suicide barrier [14]. Together, the multidisciplinary committee addressed engineering, environmental, jurisdictional, and funding challenges. Community members voiced concerns over repeated bridge closures caused by suicide, which disrupted traffic. An in-person bridge tour and stakeholder discussions increased awareness of this urgency. The trauma surgeon on the committee emphasized the human toll early in meetings, sharing the impact of bridge suicide deaths on families. Notably, local trauma surgeon advocacy helped refocus the committee’s efforts on resolving issues and securing funding, leading to Caltrans approval for the barrier.

Our study has many limitations, including the lack of data on recidivism among survivors of jumping suicides. Another concern is that advocating for a bridge barrier may be less effective due to a ‘substitution effect’, where individuals seek other means. However, the literature indicates that reducing bridge suicides at prominent locations could lead to a “displacement effect,” decreasing the number of suicides at nearby structures or via different methods. This was observed after barrier interventions on the Clifton Suspension Bridge in Bristol, England, and the Bloor Street Viaduct in Toronto, Ontario, Canada [15, 16]. A meta-analysis of suicide-prone bridge barrier installations in five countries revealed that although there was some substitution effect at nearby bridges, the overall number of suicide deaths from bridge jumps declined [11]. Our assumption of cost-effectiveness may be limited by a barrier that is not 100% effective, such as that seen with the new SFGGB grid net, or because those prevented from bridge suicide may have other issues that reduce quality of life, lifespan, and productivity or increase healthcare costs [17]. 

Future surgeon-led health policy advocacy will build on the lessons learned. Opportunities include educating the public and policymakers about the impact of loss of life and moral distress on healthcare workers, as well as about substitution and displacement effects related to suicide. Increasing awareness of community challenges, such as aesthetic, structural, and financial objections to physical suicide deterrents, is also essential. We demonstrated a financial case for cost-effective deterrents that are visually acceptable. Using data from trauma registries and other health databases can strengthen advocacy. Surgeons and organizations can promote legislation such as H.R.3505 with local and regional data at the federal and state levels.

In conclusion, an ineffective, even if well-intentioned, suicide deterrent intervention without expert involvement led to increased costs and no reduction in human death and disability. After installing the bird spike deterrent, suicide rates over the next five years did not differ significantly from the overall county trend for fall-related suicides. This study revealed that the bird spike intervention did not significantly affect bridge-related suicides. The example of the SFGGB demonstrates that a cost-effective bridge barrier could save lives. Ongoing advocacy by trauma surgeons and their allies with state and regional policymakers continues, with increasing hope that effective solutions for suicide-by-jumping from the SDC bridge and other high places can be implemented.

## Data Availability

Trauma registry data are not publicly available, but a deidentified study subset is available to academic investigators upon reasonable request. The San Diego Medical Examiner data portal is available at https://data.sandiegocounty.gov/d/jkvb-n4p7.
